# Correction: Research on partial discharge signal recognition and classification of power transformer based on acoustic-VMD and CNN-LSTM

**DOI:** 10.1371/journal.pone.0339800

**Published:** 2025-12-26

**Authors:** Liang Chen, You Wen, Huaquan Su, Ke Xiong, Danni Chen

The fifth author’s name is spelled incorrectly. The correct name is: Danni Chen. The correct citation is: Chen L, Wen Y, Su H, Xiong K, Chen D (2025) Research on partial discharge signal recognition and classification of power transformer based on acoustic-VMD and CNN-LSTM. PLoS One 20(11): e0335447. https://doi.org/10.1371/journal.pone.0335447.
